# Investigation of the Compressive Strength and Void Analysis of Cement Pastes with Superabsorbent Polymer

**DOI:** 10.3390/polym16141970

**Published:** 2024-07-10

**Authors:** Nilam Adsul, Su-Tae Kang

**Affiliations:** 1 Department of Civil Engineering, Daegu University, Gyeongsan 38453, Republic of Korea; 2 Department of Architecture Engineering, Daegu University, Gyeongsan 38453, Republic of Korea

**Keywords:** cement paste, superabsorbent polymer, water-to-cement ratio, compressive strength, water curing, relative humidity, air void analysis, air content, specific surface area, image analysis technique

## Abstract

This study aimed to experimentally investigate the compressive strength and air voids of cement pastes with varying dosages of Superabsorbent Polymer (SAP) and water-to-cement (w/c) ratios. Cement pastes were prepared using three different w/c ratios of 0.4, 0.5, and 0.6, along with different dosages of SAP ranging from 0.2% to 0.5% by weight of cement. Additionally, SAP was introduced in two forms: dry and wet. After casting the cubes, two distinct curing conditions were employed: curing at a temperature of 20 °C with a Relative Humidity (RH) of 60% (Curing 1), and water curing (Curing 2). The results revealed that the addition of SAP increased early strength when subjected to Curing 1, followed by a decrease in later strength. On the other hand, samples with SAP and water curing exhibited higher strength compared to those without SAP, especially with w/c ratios of 0.4 and 0.5. However, at a w/c ratio of 0.6, nearly all samples showed a reduction in strength compared to those without SAP. Furthermore, air void analysis was performed on all samples cured for 28 days using an image analysis technique. The samples containing wet SAP resulted in a higher total air content compared to the samples with dry SAP. Additionally, the incorporation of wet SAP in cement paste led to lower specific surface areas and a higher spacing factor than the samples with dry SAP. These findings suggest that the clumping of wet SAP particles during presoaking resulted in coarser air voids compared to the samples containing dry SAP.

## 1. Introduction

Philleo was the first to propose the idea of internal curing as a way to improve curing and lower the risk of cracking in high-performance concrete [1]. It is also used to mitigate self-desiccation and autogenous shrinkage in high-strength/performance concrete [2,3]. According to RILEM TC-196, there are two types of internal curing: (1) internal water curing (water entrainment), where the curing agent acts as a water reservoir that gradually releases water, and (2) internal sealing, where the curing agent is intended to prevent or delay water loss from the hardening concrete [2]. Many researchers have discovered various types of internal curing materials, including light-weight aggregates [4], natural fibers [5], polymer balls [6], recycled ceramic aggregate, bottom ash, and super absorbent polymers (SAPs) [3].

Zhutovsky et al. (2002) [4] used light-weight aggregate (pumice sand) in three different sizes (0.15 mm to 1.18 mm, 1.18 mm to 2.36 mm, 2.36 mm to 4.75 mm) to study the effectiveness of these aggregates in internal curing for eliminating autogenous shrinkage in HSC mixtures. The highest efficiency of internal curing was achieved with the pumice aggregate of larger size (2.36 mm to 4.75 mm), which had an absorption of 26.7%. The smaller size aggregates, having smaller absorption values, proved to be less effective. Antico et al. (2021) [5] used pig hair fibers with a diameter of 0.16 mm, added in a dry form at 2, 4, and 8 kg per m^3^ of mortar, as an internal curing material. An optimal dosage of 2 kg/m^3^ was found to reduce porosity and improve the degree of hydration. Polymer balls were also considered for internal curing, added at 2.5% and 5% by weight of cement in the concrete. The addition of 2.5% polymer balls showed excellent strength, improved durability, reduced plastic shrinkage cracking, and promoted self-healing of the concrete [6].

Similarly, SAPs can absorb and retain liquid multiple times their weight. The effectiveness of their absorption and desorption properties depends on particle size, solution properties, temperature, and humidity [7,8]. SAPs in concrete serve as internal water reservoirs and work well in mitigating self-desiccation and autogenous shrinkage compared to other materials [9,10]. SAPs can be added as a dry admixture, allowing for flexible design of pore shapes and sizes in the hardening concrete. Post-water release, SAPs create pores that help with freeze–thaw resistance but may reduce compressive strength [3,11]. However, the water released by SAPs can produce more hydration products to form a denser [12,13] and more uniform cementitious matrix to offset the strength loss [3,8,14].

Several studies have shown that the addition of SAP can reduce compressive strength [3]. This reduction becomes more pronounced as the dosage of SAP increases [14,15,16]. The decrease in strength is attributed to the increased number of pores generated by SAP within the cementitious matrix [14,16]. It is also due to the increase in the total water-to-binder (w/b) ratio caused by the additional internal curing water [16]. Therefore, the quantity of SAP, its particle size, and the amount of internal curing water provided by SAP are critical factors in influencing the strength of concrete [3].

Despite these findings, the impact of SAP on compressive strength is not invariably negative. Mönnig (2009) observed that concrete containing SAP displayed a continuous increase in strength over time, possibly due to the effects of internal curing or a denser microstructure [17]. On the other hand, Niu et al. (2024) [18] reported a decrease in strength of cement paste with SAP, which they attributed to the formation of pores due to shrinkage after release of the absorbed water. The variability in compressive strength appeared to depend on moisture conditions during curing. Conversely, the inclusion of SAP can enhance compressive strength under short curing times and dry conditions. Li et al. (2024) [19] also found that, although SAP reduced compressive strength in a standard curing environment, an increase in strength was observed when samples were cured in sealed and dry conditions.

The methodology of incorporating SAP into concrete, whether added in dry form or pre-soaked, significantly affects its strength characteristics. When dry SAP is added to mortar, there is a notable increase in compressive strength at 28 days, ranging from 10% to 50%, compared to mortar modified by pre-soaked SAP [20]. Conversely, as per a study by Kalinowski et al. (2020), pre-soaked SAP resulted in higher average compressive strength than reference and dry SAP mixtures. This is attributed to saturated SAP tending to shatter during mixing, resulting in a reduction in SAP particle size, an increase in surface area, and a more homogeneous distribution throughout the concrete [21].

Additionally, the introduction of SAP is observed to increase the volume of small capillary pores (under 20 µm) and large capillary pores (over 100 µm) within the concrete. This phenomenon diminishes over time [22]. The presence of SAP in concrete also results in a greater quantity of pores less than 300 µm in size compared to reference mixtures [23]. Increasing the dosage of both pre-soaked and dry SAP leads to a larger number of voids characterized by larger sizes and a reduced specific surface area (SSA) [24].

Despite these findings, further study is needed to fully comprehend the impact of smaller SAP sizes on the compressive strength and void structure of cement paste. This research aims to enhance the strength and durability of cementitious materials. In this study, polyacrylic-based SAP was added to cement paste with varying w/c ratios (0.4, 0.5, and 0.6) and SAP dosages (0.2–0.5% by weight of cement) to investigate its effects on compressive strength and the air void system. Analysis of air voids was performed using an image analysis technique. The SAP was tested in two forms—dry and wet (pre-soaked)—to examine their different impacts on void structure and strength.

## 2. Materials and Methods

**Figure 1:**
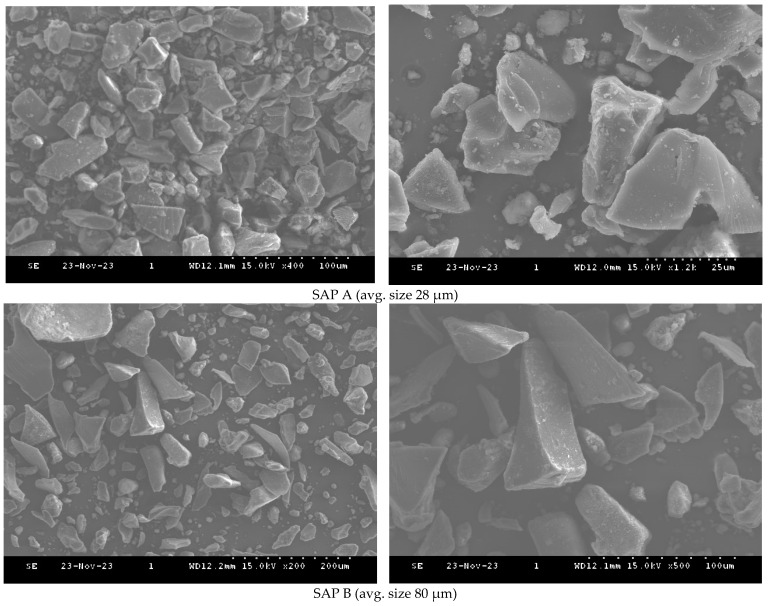
SEM images of particle sizes of SAPs.

Type 1 Portland cement from Hanil Cement in Seoul, Republic of Korea was used in this study. The cement has a density of 3.15 g/cm^3^ and a specific surface area of 2800 cm^2^/g. The chemical composition of the cement can be found in Table 1. Two sizes of commercially available polyacrylic-based SAP (TPY Co. Ltd., Seoul, Republic of Korea) were utilized: SAP A (avg. size 28 µm-TPY 900) and SAP B (avg. size 80 µm-TPY 502). Scanning Electron Microscope (SEM) (S-4300, Hitachi Ltd., Tokyo, Japan) images are shown in Figure 1. The absorption capacity of the SAPs was tested according to RILEM guidelines (2018) [25]. SAP A exhibited an absorption capacity of 20.71 g/g, while SAP B had a capacity of 17.70 g/g after 30 min, as determined using the tea-bag method in a cement filtrate solution. Furthermore, the filtration method revealed that SAP A had an absorption capacity of 36.82 g/g, and SAP B had an absorption capacity of 35.62 g/g after 30 min in the cement filtrate solution. For this study, cement pastes were prepared using different w/c ratios (0.4, 0.5, and 0.6) and various SAP dosages (0.2%, 0.3%, 0.4%, and 0.5% by weight of cement), for a total of 48 mixes. A w/c ratio of less than 0.4 was found to produce a highly viscous mixture during the experimental examination, making homogeneous mixing and subsequent testing more difficult. As a result, w/c ratios greater than 0.4 were therefore selected.

The dry SAP samples were labelled with a ‘D’ prefix, while the wet SAP samples had a ‘W’ prefix. The labels ‘A’ and ‘B’ were used to distinguish between the two variations of SAP. For example, mixes with dry and wet SAP types A and B, with a w/c ratio of 0.4 and SAP dosages ranging from 0.2 to 0.5%, were designated as D-1A to D-4A and D-13B to D-16B, as well as W-25A to W-28A and W-37B to W-40B. Similarly, mixes with dry and wet SAP type A and B, featuring a w/c ratio of 0.5 and SAP dosages ranging from 0.2 to 0.5%, were labelled as D-5A to D-8A, D-17B to D-20B, W-29A to W-32A, and W-41B to W-44B. Lastly, mixes containing both dry and wet SAP type A and B, with a w/c ratio of 0.6 and a range of SAP dosages from 0.2 to 0.5%, were designated as D-9A to D-12A, D-21B to D-24B, W-33A to W-36A, and W-45B to W-48B. Some of the sample mixes are described in Table 2.

For the cement paste modified with dry SAP, the cement and dry SAP were mixed for one minute in a Hobart mixer at speed 2 (95 RPM). Water was then added, and the mixture was blended for another 30 s. The mixer was paused to scrape the paste from the walls and bottom of the mixing bowl, which took approximately 50 s. Afterwards, the paste was mixed again at the same speed for 40 s and finally blended at speed 4 (135 RPM) for one minute. 

For the wet SAP preparation, dry SAP was mixed with water and left to pre-soak for 30 min before being added to the dry cement. The cement was first mixed alone at speed 2 (95 RPM) to remove any clumps. Then, the SAP–water mixture was incorporated into the cement and mixed at the same speed for one minute. The mixer was stopped, and the sides of the bowl were scraped for about 30 s. Mixing resumed for another 60 s at speed 2 (95 RPM), followed by scraping the bottom of the bowl once again. The final step involved mixing the paste for 60 s at speed 4 (135 RPM).

During the preparation and casting of the cement paste, room conditions were consistently maintained at a temperature of 20 ± 2 °C and a relative humidity of 30 ± 5%. To ensure uniformity, the water used in the preparation of the cement paste was held at a temperature of 20 °C using a water bath from Joanlab Equipment Co., Ltd., Huzhou City, China.

**Figure 2:**
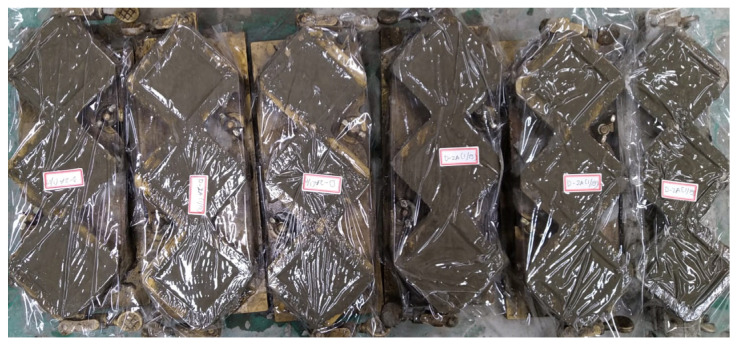
Sample casting and covering with thin plastic film.

For each cement paste mixture, a total of 18 cubes, each measuring 50 mm × 50 mm, were cast, as shown in Figure 2. Two curing conditions were applied in this study: curing at a temperature of 20 °C and 60% RH (Curing 1) and water curing (Curing 2). All the samples were covered with thin plastic film immediately after casting to prevent evaporation of surface water. Samples intended for water curing were first kept under the same conditions as curing 1 for 24 h, at a temperature of 20 °C and 60% RH, to dry. After this period, the samples were demolded, and nine cubes from each batch were submerged in water for curing until testing.

**Figure 3:**
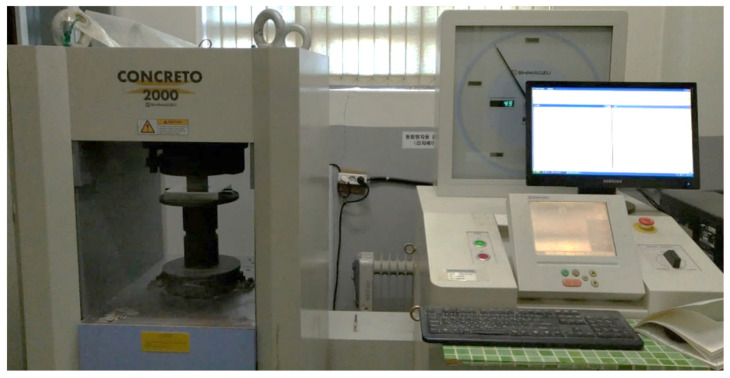
Compressive strength test on samples.

A compressive strength test was performed on the hardened cement paste samples at intervals of 3, 14, and 28 days, using a compression testing machine (Shimadzu Corporation, Kyoto, Japan), as depicted in Figure 3.

**Figure 4:**
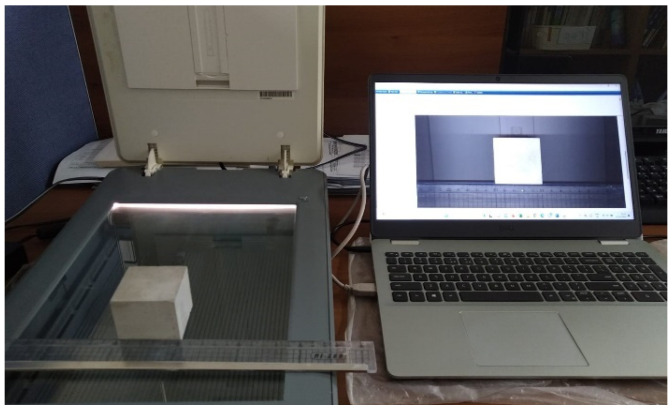
Sample surface scanning by flatbed scanner.

To conduct air void analysis, the cement pastes cubes (50 mm × 50 mm) cured for 28 days were trimmed by 10 mm on two sides. Subsequently, these two sides were scanned using a flatbed scanner (HP Scanjet G4010, Irvine, CA, USA) at a resolution of 1200 dpi, as shown in Figure 4. The scanned images were then analyzed using ImageJ software (version 1.54h) for image analysis.

## 3. Results and Discussion

### 3.1. Compressive Strength

**Figure 5:**
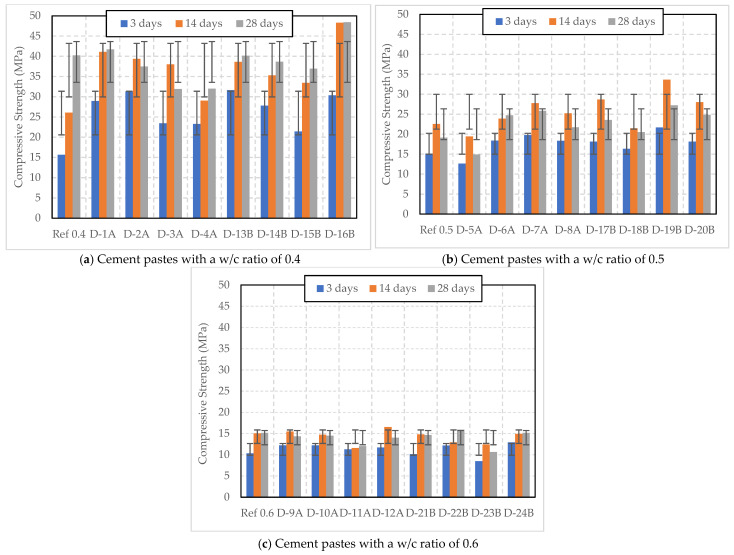
Compressive strength of cement pastes with w/c ratios of 0.4, 0.5, and 0.6 and varying dosages of dry SAP, cured under Curing 1 conditions (temperature of 20 °C and 60% RH).

Figure 5 presents the compressive strength of cement pastes with different w/c ratios (0.4, 0.5, and 0.6) and varying dosages of dry SAP A and B (0.2–0.5% by weight of cement), cured under Curing 1 conditions. In Figure 5a, it is evident that the addition of dry SAP decreased the strength compared to the reference sample with a w/c ratio of 0.4 in most of the samples. However, samples with 0.2% SAP A (D-1A), 0.2% SAP B (D-13B), and 0.4% SAP B (D-16B) exhibited higher strength than the reference sample without SAP. Specifically, D-1A recorded an early compressive strength of 28.93 MPa at 3 days that slightly increased to 41.73 MPa at 28 days. Notably, D-16B showed exceptional strength growth compared to the other samples, with an initial strength of 30.35 MPa at 3 days and an outstanding final strength of 48.4 MPa at 28 days, indicating that a 0.5% dosage of SAP B significantly enhances the cement’s compressive strength over time under Curing 1 conditions. On the other hand, D-13B initially reached 31.6 MPa but had a slightly lower strength of 40.13 MPa at 28 days compared to the reference. When a 0.3% SAP dosage was used, D-2A showed higher initial strength compared to D-14B, but its strength decreased over time, while that of D-14B consistently increased. Similarly, D-3A had higher initial strength than D-15B, but lower strength after 28 days. Higher dosages of SAP A above 0.3% led to a reduction in compressive strength compared to the reference sample. This aligns with a study by Huang et al. (2022) [26], which found that, as the SAP dosage increased, the compressive strength initially increased and then decreased. This may be attributed to water absorption by SAP during mixing, which reduces the effective w/c ratio and increases strength. Additionally, the voids (macro-pores) created by the water absorbed by the SAP can further decrease the compressive strength [3,26].

As the w/c ratio increased, the compressive strength decreased. In Figure 5b, the cement pastes with a w/c ratio of 0.5 and cured under Curing 1 conditions showed a reduction in 28-day compressive strength. This strength was lower than the 14-day compressive strength for all samples, except for D-6A, which showed an increase from 18.39 MPa at 3 days to 23.89 MPa at 14 days, and 24.73 MPa at 28 days. D-5A with the lowest dosage of SAP A exhibited the weakest performance, even lower than the reference sample, with a 28-day strength of 14.93 MPa. D-7A, with 0.4% SAP A, showed a higher initial strength of 19.74 MPa and the highest 14-day strength of 27.78 MPa, which decreased to 25.85 MPa at 28 days. In contrast to this study, Beushausen et al. (2014) [27] found that adding SAP to mortar with a water-to-binder ratio of 0.55 led to a slight decrease in early-age compressive strength, which partially recovered over time with internal curing.

Among the samples with SAP B, D-19B with a 0.4% dosage exhibited the highest initial strength of 21.65 MPa and the highest 14-day strength of 33.65 MPa, across all samples with SAP B, but this decreased to 27.23 MPa at 28 days. Conversely, D-18B with a 0.3% dosage of SAP B showed lower strengths at all stages compared to other mixes with SAP B.

In Figure 5c, it can be observed that the cement pastes with a w/c ratio of 0.6 and varying dosages of SAP A under Curing 1 conditions showed lower compressive strength compared to the reference sample. D-22B and D-24B with 0.3% and 0.5% SAP B dosages, respectively, exhibited higher 28-day compressive strength of 15.9 MPa and 15.18 MPa compared to the reference sample strength of 15.14 MPa. D-21B showed a minimal reduction in compressive strength compared to the reference sample. On the other hand, D-23B with a 0.4% SAP dosage showed the lowest compressive strength among all the samples.

**Figure 6:**
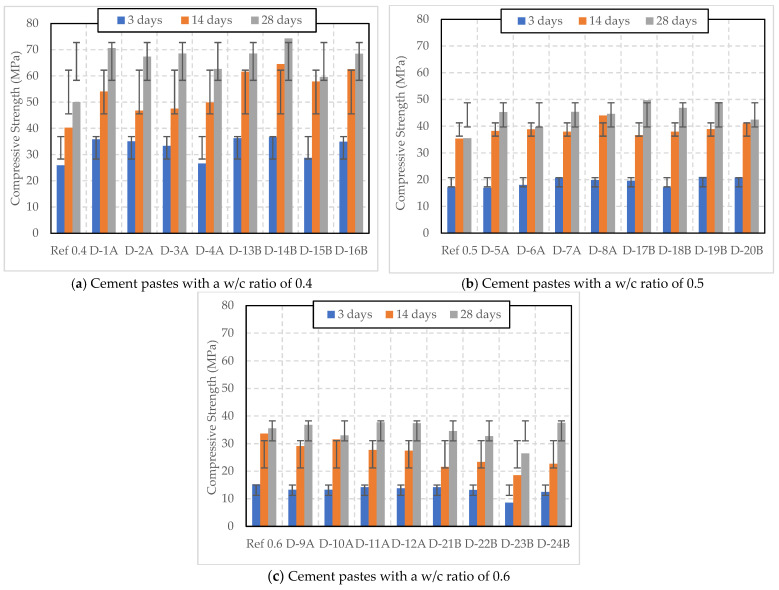
Compressive strength of cement pastes with w/c ratios of 0.4, 0.5, and 0.6 and varying dosages of dry SAP, cured under Curing 2 condition (water curing).

From Figure 6a, it can be observed that mixes with a w/c ratio of 0.4 and cured under Curing 2 conditions exhibited increased compressive strength for all samples containing SAP A and SAP B, compared to the reference sample without SAP. The reference sample achieved strengths of 25.90 MPa, 40.28 MPa, and 50.03 MPa at 3, 14, and 28 days respectively. Incorporating 0.2% SAP A (D-1A) significantly increased strengths to 35.81 MPa, 54.05 MPa, and 70.59 MPa at the respective ages. However, a higher dosage of 0.3% SAP A (D-2A) did not appreciably increase the 28-day strength compared to the 0.2% dosage, but showed lower strengths at earlier ages. With 0.4% SAP A (D-3A), the strengths were further reduced at early ages compared to the 0.2% dosage but slightly increased at 28 days. The higher dosage of 0.5% SAP A (D-4A) resulted in early strength loss, such as 26.63 MPa (at 3 days) and 49.85 MPa (at 14 days), with only a marginal increase at 28 days (62.6 MPa) compared to D-1A. Similarly, increasing strengths were observed with 0.2% and 0.3% SAP B (D-13B and D-14B), achieving the highest 28-day strengths of 68.54 MPa and 74.25 MPa, respectively. However, a higher dosage of 0.4% SAP B (D-15B) and 0.5% SAP B (D-16B) reduced compressive strength at all ages compared to the D-13B and D-14B samples.

Both dry SAP A and SAP B led to increased compressive strength at optimal dosages. SAP B dosed at 0.3% (D-14B) resulted in the highest 28-day compressive strength observed, outperforming all SAP A and B dosages. While some SAP additions can improve strength, they must be dosage-constrained, because excessive SAP can reduce compressive strength at certain curing ages.

Figure 6b presents the compressive strengths of cement paste samples with a w/c ratio of 0.5, incorporating varying dosages of SAP and cured under Curing 2 conditions. The reference sample without SAP displayed compressive strengths of 17.13 MPa (3 days), 35.36 MPa (14 days), and 35.48 MPa (28 days), respectively. All samples containing SAP performed well compared to the sample without SAP.

Adding 0.2% SAP A (D-5A) led to a slight decrease in early strength at 3 days (16.96 MPa), but eventually showed improvement over the reference sample at later ages, with the 28-day strength reaching 45.24 MPa. Increases at 0.3% SAP A (D-6A) were marginal across all ages, while the more considerable increase in early strength at 3 days for the 0.4% SAP A (D-7A) dosage was substantial, maintaining a higher 28-day strength of 45.36 MPa as well. The 0.5% SAP A dosage (D-8A) also showed improvement over the reference at 14 and 28 days, but this was less pronounced than the 0.4% dosage.

When considering SAP B, even the lowest dosage of 0.2% (D-17B) demonstrated a significant increase in both early and late strengths, reaching a peak of 49.67 MPa at 28 days. A dosage of 0.3% (D-18B) resulted in a strength increase at 28 days, although it was lower than the 0.2% dosage. The highest early strength of 21.04 MPa was exhibited by the 0.4% dosage (D-19B), and it also had an increased strength of 49.01 MPa at 28 days, which was close to that of the 0.2% dosage. However, the 0.5% dosage (D-20B) did not achieve this maximum, especially at 28 days. The effectiveness of SAP additions appeared to peak at different dosage levels for SAP, with SAP B at 0.2% and 0.4% dosages resulting in the highest 28-day strength.

From Figure 6c, it can be observed that the SAP A series (D-9A to D-12A), ranging from 0.2% to 0.5% dosages, showed a slight decrease in early strength at 3 days compared to the reference. However, all dosages showed some improvement in strength at 28 days, with 0.4% SAP A showing the highest increase to 37.74 MPa.

For the SAP B series (D-21B to D-24B), the 0.2% dosage showed a slight reduction in strength at 14 days but improved at 28 days (34.55 MPa), although slightly less than the reference. Increasing the SAP B dosage to 0.3%, 0.4%, and 0.5% resulted in further reductions in early and middle-age strengths. Remarkably, the 0.5% dosage of SAP B (D-24B) did show a notable increase at 28 days, finishing with the highest strength of 37.53 MPa among all SAP B samples. Overall, while initial strengths were generally lower for paste samples containing SAP, several configurations displayed improved 28-day compressive strengths, suggesting a nuanced relationship between SAP dosage and cement strength development, with potential benefits at certain dosages and curing times.

Overall, it is clear that the use of dry SAP in cement paste samples, cured under Curing 1 conditions, generally resulted in a decrease in compressive strength in most cases. In contrast, under Curing 2 conditions (water curing), a consistent increase in strength was observed in many cases, suggesting that water curing effectively enhanced the strength of dry SAP-modified cement pastes.

**Figure 7:**
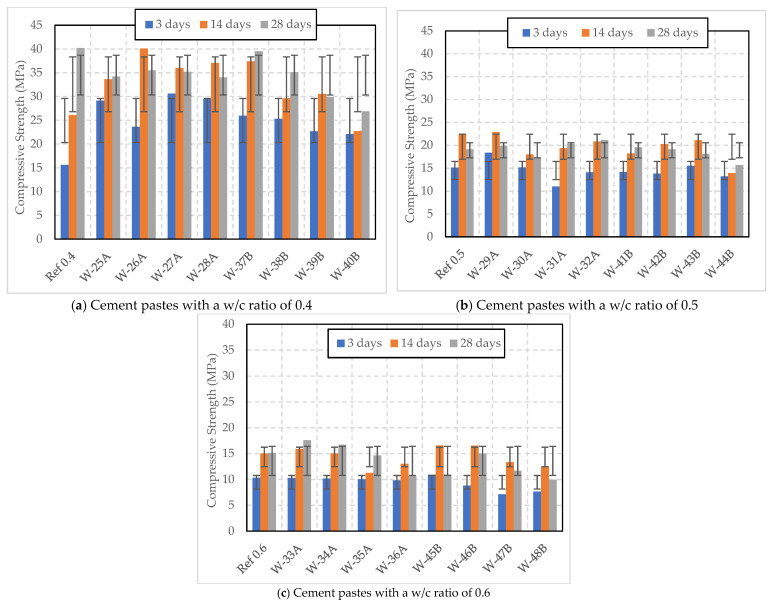
Compressive strength of cement pastes with w/c ratios of 0.4, 0.5, and 0.6 and varying wet SAP dosages cured under Curing 1 condition (temperature of 20 °C and 60% RH).

**Figure 8:**
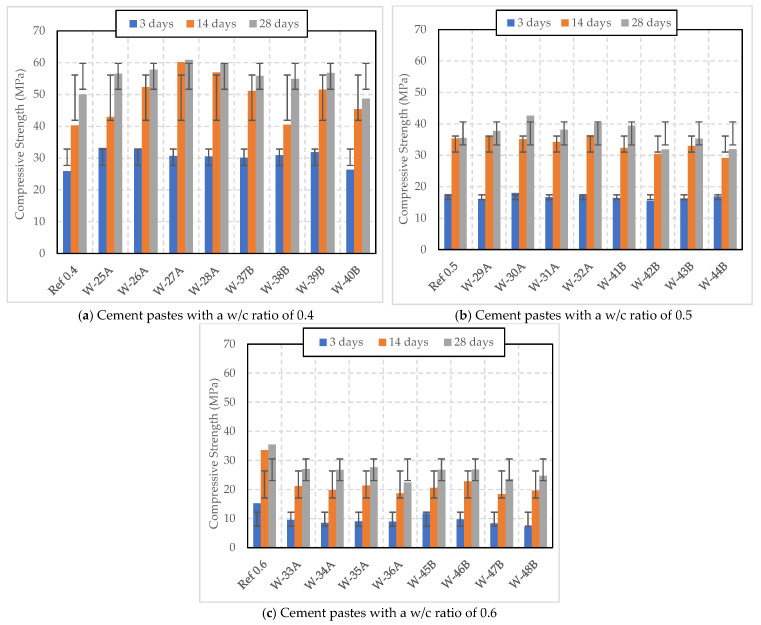
Compressive strength of cement pastes with w/c ratios of 0.4, 0.5, and 0.6 and varying dry SAP dosages, cured under Curing 2 conditions (Water Curing).

Figure 7 and Figure 8 illustrate the compressive strength of cement pastes with varying w/c ratios and dosages of wet SAP, cured under Curing 1 and Curing 2 conditions. In Figure 7a, all samples with wet SAP A exhibited a reduction in compressive strength compared to the reference. The inclusion of wet SAP A helped increase strength at 3 and 14 days compared to the reference, but it had an impact on the 28-day strength development.

On the other hand, the introduction of wet SAP B resulted in a reduction in compressive strength as the SAP dosage increased from 0.3%. At a dosage of 0.2% SAP B, the sample surpassed the reference with a 28-day strength of 39.53 MPa, slightly lower than the reference. However, the inclusion of wet SAP B led to a continuous increase in strength, which was contrary to the results observed with SAP A.

In Figure 7b, at a dosage of 0.2% SAP, sample W-29A exhibited strengths of 18.35 MPa at 3 days, 22.89 MPa at 14 days, and 19.89 MPa at 28 days. In contrast, W-41B showed strengths of 14.16 MPa at 3 days, 18.19 MPa at 14 days, and 19.56 MPa at 28 days. This indicates that SAP A leads to higher early strength development than SAP B, although by 28 days the strengths are relatively similar. W-41B showed continuous development in strength, which was opposite to W-29A. However, higher doses of SAP A, such as 0.3% and 0.4% (W-30A and W-31A), resulted in lower early strengths at 3 and 14 days. Nevertheless, the 0.4% SAP A sample slightly surpassed the reference at 28 days with a strength of 20.3 MPa. Higher doses of wet SAP A led to a slight increase in compressive strength over time, reaching 21.13 MPa at 28 days compared to the reference. Sample W-43B demonstrated higher initial strength development, starting at 15.51 MPa at 3 days and ending at 18.14 MPa at 28 days, which was lower than W-31A and the reference. On the other hand, W-44B had the lowest strengths among all measured ages, compared to the other samples. According to a study conducted by Kong et al. (2014), the inclusion of pre-soaked SAP marginally diminished compressive strength, particularly noticeably in early-age concrete. However, this can be mitigated by selecting an appropriate dosage of SAP [28].

In Figure 7c, both W-33A and W-34A initially had strengths comparable to the reference, but surpassed it by 28 days, reaching 17.59 MPa and 16.71 MPa, respectively, indicating better performance over time. W-35A (0.4% dosage) strength started just below the reference and showed a significant drop at 14 days before slightly recovering to 14.63 MPa at 28 days. W-36A, which contained a higher dosage (0.5%), consistently had lower strength throughout the testing period, with the lowest 28-day strength among all SAP A samples at 10.69 MPa.

All the samples containing SAP B resulted in a decrease in strength over time compared to samples with SAP A and the reference. Sample W-45B exhibited higher early strength than the reference, peaking impressively at 16.58 MPa at 14 days but experiencing a sharp decline to 10.96 MPa at 28 days. W-46B (0.3% dosage) had the lowest initial strength but demonstrated substantial gain by 14 days; however, it decreased to 14.98 MPa at 28 days. The higher dosage of SAP B, W-47B and W-48B, showed lower compressive strength at all intervals compared to the other samples.

Figure 8 demonstrates that the addition of wet SAP to cement paste, cured under Curing 2 conditions, enhances compressive strength compared to the reference sample without SAP. In Figure 8a, W-25A with 0.2% wet SAP A consistently surpasses the reference sample at all stages, indicating the beneficial effect of this SAP on compressive strength; it reached a strength of 56.55 MPa at 28 days. W-26A shows the most significant increase in strength over time, starting at 32.96 MPa and reaching the highest strength of 57.79 MPa at 28 days. The SAP A series (W-25A to W-28A) demonstrates a trend where compressive strength improves with increasing SAP dosages up to 0.4%. W-27A achieved the highest 28-day strength at 60.89 MPa. W-28A shows a slight decline compared to the 0.4% dosage but still outperformed the reference sample, reaching 59.88 MPa at 28 days.

W-37B exhibited higher initial strength compared to the reference and maintained higher strength at 28 days, reaching 55.89 MPa. W-38B and W-39B, with 0.3% and 0.4% wet SAP B, respectively, showed increased strength over time, while W-40B with 0.5% exhibited the lowest compressive strength at 28 days among the wet SAP B series and the reference sample.

Figure 8b shows that the addition of wet SAP A leads to an increase in strength over time, while wet SAP B results in a decrease with increasing dosage. W-29A initially had lower 3-day strength than the reference but slightly surpassed it with a strength of 37.79 MPa at 28 days. W-30A had higher initial strength than the reference that continued to develop, resulting in the highest strength at 28 days (42.63 MPa) among all samples. W-31A started with lower 3-day strength but then followed an upward trend, reaching a 28-day strength of 38.16 MPa. W-32A maintained high strength throughout the test period, ending with a robust 40.26 MPa at 28 days, consistently improving over the reference sample.

W-41B initially started slightly below the reference in terms of initial strength but exhibits the highest 28-day strength of 39.4 MPa among the SAP B series. W-42B (0.3% dosage) has lower strength at all stages compared to the reference, with a notable drop, concluding with 31.86 MPa at 28 days. W-43B shows an increasing trend in strength, although reaching only 35.25 MPa at 28 days, slightly below the reference. The sample W-44B (0.5% dosage) reflected improvement between 3 and 14 days but then displayed decreasing strength, ending with 31.9 MPa at 28 days, the lowest overall among the SAP B samples.

From Figure 8c, it can be observed that all cement paste samples with a w/c of 0.6 and SAP displayed lower compressive strengths throughout the curing process compared to the reference sample without SAP. This indicates that higher dosages of wet SAP A & B and a higher w/c ratio may have a negative impact on strength development. Interestingly, within the SAP A series, samples W-33A and W-35A achieved the highest 28-day strengths, at 27.03 MPa and 27.59 MPa, respectively. However, these strengths were still lower than that of the reference sample. Notably, the sample with the highest SAP A dosage, W-36A (0.5% SAP A), consistently exhibited the lowest compressive strengths throughout all testing periods. This suggests that higher dosages of SAP A do not necessarily result in better strength outcomes when samples are prepared with a w/c ratio of 0.6 and cured under Curing 2 conditions.

Similarly, like SAP A, all SAP B samples demonstrated lower compressive strength values compared to the reference sample at all recorded times. Among the SAP B series, W-46B (0.3% SAP B) achieved the highest 28-day strength at 26.9 MPa. However, this value still falls below that of the reference sample. W-48B (0.5% SAP B) showed a trend of increasing compressive strength over time but consistently fell short of the reference sample at each interval, with a 28-day strength of 24.73 MPa. This indicates that a higher content of SAP B may not be beneficial in terms of strength development.

The observed differences in performance among the samples suggest that the dosage of SAP and the curing conditions have a significant impact on the development of compressive strength. Therefore, it is crucial to optimize the SAP dosage and curing conditions to achieve the desired strength characteristics in cement paste. Cement paste cured under Curing 1 conditions (temperature of 20 °C and 60% RH) resulted in a decrease in strength after 14 days. However, the use of Curing 2 conditions (water curing) yielded positive results when used with cement paste containing SAP A and SAP B. This is in contrast to a study conducted by Sun et al. (2019), which found that water curing decreased strength, while air curing enhanced the later-age strength of cement mortar and concrete [15]. Furthermore, the inclusion of dry SAP resulted in higher compressive strength development compared to the addition of wet SAP. Similar findings were reported by Tan et al. (2019), who discovered that the addition of dry SAP increased the 28-day compressive strength by approximately 10–50%, whereas wet SAP resulted in a strength increase ranging from -26% to 6% [20].

### 3.2. Void Analysis by ImageJ

The scanned images of the samples cured for 28 days were processed using ImageJ software (version 1.54h). Two images were processed for each sample. The specific surface area (SSA) and spacing factor were then calculated based on the equations suggested by [29,30,31]. The specific surface of air voids in concrete measures their total surface area relative to their volume. According to ASTM C457 [32], a specific surface range of 23.6 to 43.3 mm^−1^ is recommended for concrete to resist damage from freeze–thaw cycles. Higher values indicate a finer distribution of air voids [31,32]. The spacing factor, defined by Power, is a parameter related to the maximum distance of any point in the cement paste from the periphery of an air void [29]. It is the primary indicator of the air void system’s ability to withstand frost attack, with around 0.200 mm suggesting effective frost resistance [29,31]. Furthermore, the air content is equal to the percentage of area occupied by air voids, as obtained from image analysis [29].

Table 3, Table 4, Table 5, Table 6, Table 7 and Table 8 depict the void analysis in cement paste with varying w/c ratios and SAP dosages (dry and wet) cured under Curing 1 and Curing 2 conditions. From Table 3, it can be observed that cement paste with SAP A showed a range of total air content from 1.81% to 2.41%, while that of paste with SAP B ranged from 1.70% to 2.65%. Sample W-40B (0.5% SAP) exhibited the highest total air content of 2.65%. On the other hand, samples W-27A (0.4% SAP) and W-28A (0.5% SAP) exhibited significantly lower percentages of air voids under 1 mm, suggesting a smaller proportion of fine air bubbles and a higher proportion of larger bubbles compared to other samples. Generally, the SSA was higher in most of the samples with dry SAP A compared to SAP B; samples D-3A and D-15B had particularly high SSAs. In samples with wet SAP A, the SSA was lower than in samples with wet SAP B. Cement paste containing dry SAP A cured under Curing 1 conditions tended to have lower spacing factors overall, indicating closer spacing of air voids, which could have impacted the performance characteristics of the cement paste. Overall, samples with dry SAP A tended to have slightly higher air content, higher SSA, and lower spacing factors compared to samples with dry SAP B. On the other hand, samples with wet SAP A resulted in higher air content, higher spacing factors, and lower SSA compared to samples with wet SAP B. Additionally, wet SAP resulted in higher total air content compared to dry SAP mixes.

**Figure 9:**
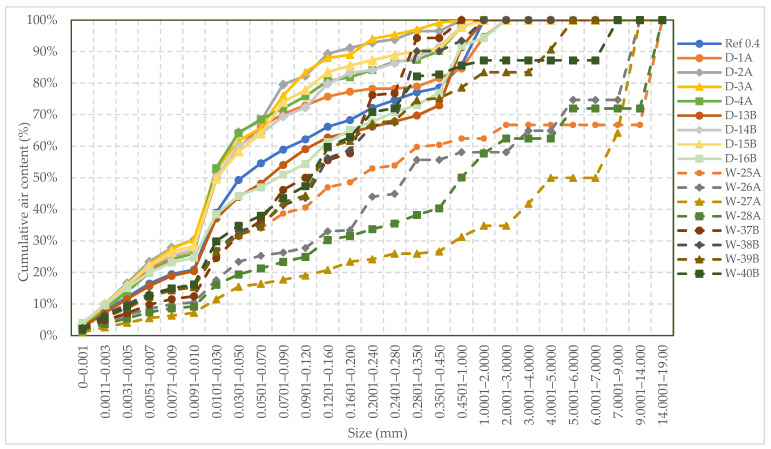
Cumulative air content in cement pastes with a w/c ratio of 0.4 and wet and dry SAP cured under Curing 1 conditions.

Figure 9 illustrates the cumulative air content in cement paste with a w/c ratio of 0.4. The paste contained different dosages of wet and dry SAP A and B and was cured under Curing 1 conditions. It is observed that the paste containing wet SAP has larger voids exceeding 1 mm in size. These larger voids are mostly observed in mixes with SAP A, which can be explained by the gel-blocking effect. This effect occurs when fine SAP particles (size less than 100 µm) come into contact with liquid, resulting in minimal absorption on their surface. As a result, the particles slightly swell and stick together, forming clusters consisting of both swollen and unswollen particles. This hinders further disaggregation and absorption [10,32,33]. On the other hand, the cement pastes with dry SAP showed a higher number of smaller voids under 1 mm compared to the cement paste with wet SAP and without SAP. Samples D-2A and D-3A have a similar void system, while D-4A has a greater number of finer voids ranging from 0.01 to 0.07 mm. Additionally, among all the samples containing wet and dry SAP, W-27A (0.4%) and W-28A (0.5%) have fewer smaller voids, but a smaller number of larger voids ranging from 1 to 14 mm are present.

**Figure 10:**
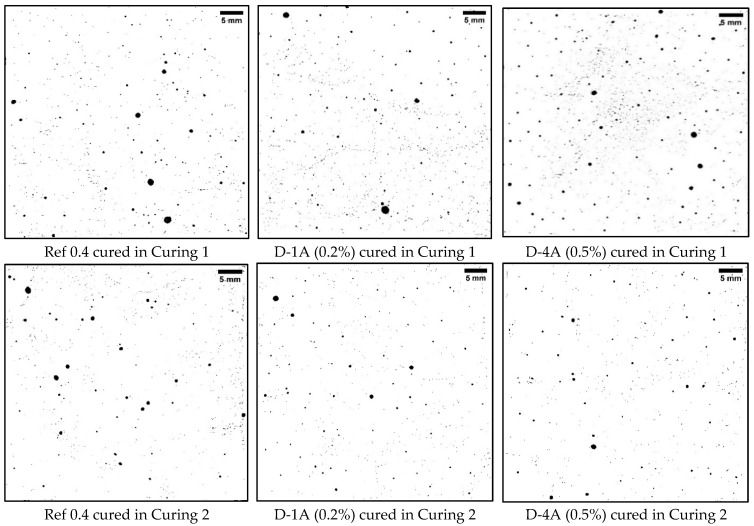
Binary images of samples prepared with dry SAP and a w/c ratio of 0.4.

**Figure 11:**
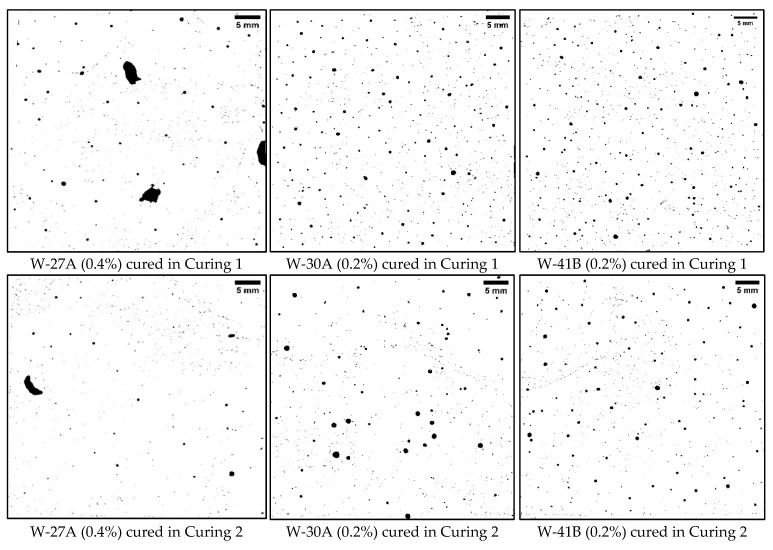
Binary images of samples prepared with wet SAP and a w/c ratio of 0.4/0.5.

Upon comparing the above findings with the data presented in Table 4, it is clear that Curing 2 (water curing) conditions led to a reduction in voids and an enhancement in strength. All samples subjected to Curing 2 conditions had an air content of less than 2%, with sample W-40B exhibiting the highest air content at 1.83% and a standard deviation of 0.09. This variation in air content suggests differences among the samples. It is worth noting that the samples with dry SAP had lower air content compared to samples with wet SAP, and the reference sample, D-1A, had the lowest air content at 0.95%. Additionally, sample D-4A, which had the highest dosage of SAP A, exhibited the highest SSA of 91.09 mm^−1^. Conversely, sample D-16B, which had a higher SAP B dosage, had the lowest SSA of 44.46 mm^−1^ among the SAP A and B samples. Furthermore, the samples with dry SAP B displayed a slightly higher spacing factor compared to the other mixes, with D-16B having the highest spacing factor at 0.25 mm. On the other hand, the samples with dry SAP A displayed a higher spacing factor compared to samples with wet SAP A. Figure 10 and Figure 11 illustrate the differences in the air void system resulting from the two curing conditions and the addition of dry and wet SAP. In the binary images, black indicates voids, while white represents the cement paste.

Figure 11 clearly illustrates the impact of Curing 1, Curing 2, and wet SAPs on the void system. The presence of wet SAP A and its gel-blocking effect can account for the larger voids observed in the W-27A sample. Furthermore, the voids in samples containing wet SAP A were significantly larger compared to those with wet SAP B.

Table 5 presents the void analysis data for cement paste with a w/c ratio of 0.5, as well as wet and dry SAP samples that were cured under Curing 2 conditions. It is evident that, among the SAP A samples, D-5A exhibits the highest total air content at 3.26%, followed closely by W-44B at 3.30% among the SAP B samples. However, the SAP A mixes resulted in lower total air content compared to the SAP B and reference mixes. Similarly, samples D-5A, D-18B, W-30A, W-41B, and W-44B have the highest percentage of air voids ≤ 1 mm, indicating the presence of finer air voids in these samples. Additionally, D-8A and W-44B have the highest SSAs among the dry and wet SAP samples, measuring at 79.05 mm^−1^ and 61.27 mm^−1^, respectively. Furthermore, D-5A and D-8A have the smallest spacing factor among the dry samples, measuring at 0.09 mm. Conversely, among the wet SAP samples, W-44B has the smallest spacing factor, measuring 0.11 mm.

Table 6 indicates void analysis data for cement paste with a w/c ratio of 0.5 and varying SAP dosages cured under Curing 2 conditions. The D-6A and W-29A samples, which contained dry and wet SAP A, exhibited the highest total air content at 2.14% and 2.15%, respectively. Comparatively, the reference sample had an air content of 2.22%. Similarly, the D-20B and W-42B samples showed the highest air content at 1.90% and 2.40%, respectively. The samples containing dry SAP and no SAP had more fine voids under 1 mm compared to the samples with wet SAP. Among the samples containing SAP A, D-8A had the highest SSA at 90.13 mm^−1^ and a lower spacing factor at 0.10 mm. The D-19B sample, on the other hand, showed a higher SSA, at 78.11 mm^−1^, among the cement pastes containing SAP B.

Table 7 presents void analysis data for cement paste with a w/c ratio of 0.6, cured under Curing 1 conditions. The air content for all the samples is above 3%, with D-23B, W-36A, and W-48B having the highest air contents, at 3.99%, 3.98%, and 4.06%, respectively, compared to the other samples. Among all the samples, W-48B showed exclusively fine voids under 1 mm in size, indicating a higher prevalence of fine voids. The D-23B samples displayed a higher SSA at 88.57 mm^−1^, while W-33A exhibited the lowest SSA at 55.91 mm^−1^. Furthermore, samples containing dry SAP showed a lower spacing factor compared to the reference and wet SAP samples.

From Table 8, it is evident that the total air content decreased in Curing 2 compared to Curing 1. The dry and wet SAP A samples had higher total air contents, at 2.58% and 3.19%, respectively, in D-10A and W-36. Among the SAP B samples, D-23B exhibited a higher total air content, at 2.95%, while W-47B had 3.17%. Samples containing 0.3% to 0.5% of dry SAP A and B showed a finer void structure under 1 mm. Among dry and wet SAP samples, D-24B and W-36A had the lowest SSAs, at 64.68 mm^−1^ and 60.80 mm^−1^, respectively. On the other hand, D-9A had the highest SSA, at 75.66 mm^−1^, among dry SAP samples, while W-33A had the highest among wet SAP samples at 63.71 mm^−1^.

Overall, it can be seen that samples with dry SAP generally had higher SSAs and smaller spacing factors compared to samples with wet SAP. This indicates that the inclusion of dry SAP in cement paste leads to more finely distributed air voids with smaller sizes, resulting in higher SSA and smaller spacing factors. However, cement pastes with wet SAP showed slightly lower SSAs and larger spacing factors, indicating coarser air voids with larger sizes, which is consistent with a study conducted by Riyazi et al. (2017) [24]. Additionally, the curing conditions, namely Curing 1 and Curing 2, had a significant impact on void structure. Samples cured under Curing 1 exhibited higher air content than those samples cured under Curing 2 (water curing).

### 3.3. Analysis of Variance (ANOVA) on Air Void Analysis Data

ANOVA provides effective analysis for comparing more than two groups. ANOVA single-factor analysis was carried out in Microsoft Excel on the air void analysis data of cement pastes with varying dosages of dry and wet SAPs and w/c ratios, as shown in Table 9, Table 10, Table 11 and Table 12. The aim of the analysis was to determine statistically significant differences between four groups: total air, air ≤ 1mm, SSA, and spacing factor.

The ANOVA analysis provided valuable insights into differences in the air void characteristics of cement pastes with different SAP dosages and w/c ratios (0.4 and 0.5), cured under two different conditions. The mean (average) of total air (%) and air ≤ 1 mm increased as the w/c ratio increased, suggesting more air voids. Similarly, the mean of SSA increased with higher w/c ratios, indicating a larger surface area of voids. Conversely, the mean of the spacing factor decreased with the w/c ratio, suggesting that air voids were closely located.

The mean square (MS) decreased as the w/c ratio increased, indicating reduced variability within groups. Furthermore, the F-statistics values were higher than the F-critical values, with negligible *p*-values, confirming that the differences between the groups were statistically significant. This demonstrates that the changes in air void properties were not random but were influenced by different SAP dosages, w/c ratios, and curing conditions. Therefore, the addition of SAPs, the w/c ratios, and the curing regime had a significant effect on the air void structure in the cement paste.

### 3.4. Relationship between 28-Day Compressive Strength and Air Content

**Figure 12:**
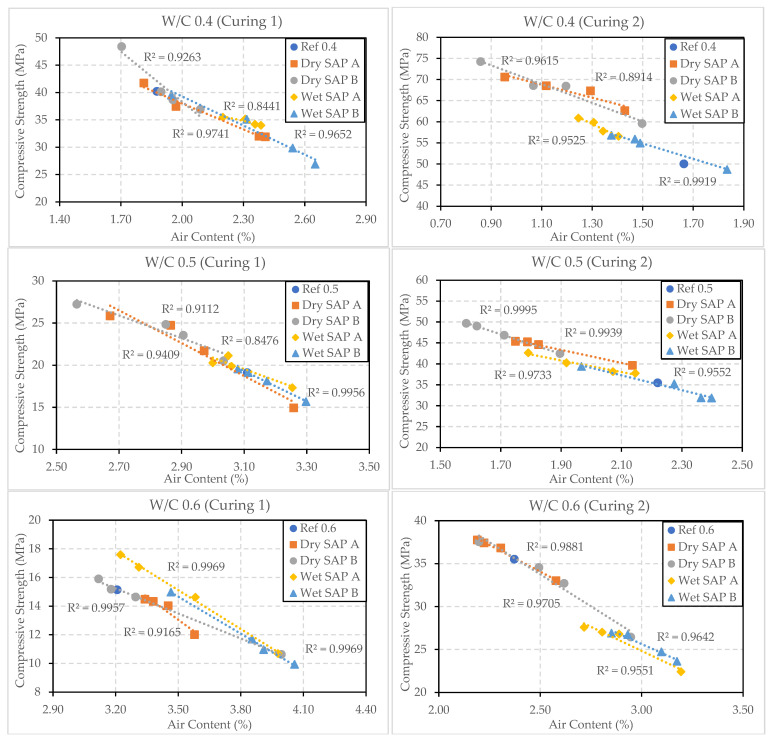
Relationship between air content and compressive strength of samples with varying w/c ratio and SAP content (dry and wet) cured under Curing 1 and Curing 2 conditions.

Figure 12 illustrates the relationship between the air content and compressive strength of samples with different w/c ratios (0.4, 0.5, and 0.6) and varying amounts of SAP A and B (wet and dry) (0.2–0.5%). It is evident that a higher air content (%) leads to decreased compressive strength, while a lower air content (%) results in higher compressive strength. Among all the samples, those with a w/c ratio of 0.4 cured under Curing 2 conditions exhibit the lowest air content and highest strength. On the other hand, samples with a w/c ratio of 0.6 cured under Curing 1 conditions showed higher air content and lower compressive strength. The addition of wet SAP resulted in higher air content and lower compressive strength compared to samples with dry SAP in all cases, except for samples with a w/c ratio of 0.6 cured under Curing 1 conditions, which showed that the addition of dry SAP resulted in lower strength and higher air content. Among samples with wet SAP, the addition of wet SAP B resulted in higher air content and lower strength. The coefficient of determination (R^2^) is slightly lower in samples with a w/c ratio of 0.4 containing SAP A in wet and dry form cured under Curing 1 and 2 conditions, at 0.84 and 0.89, respectively, compared to other samples under similar conditions.

### 3.5. Microstructural Analysis

**Figure 13:**
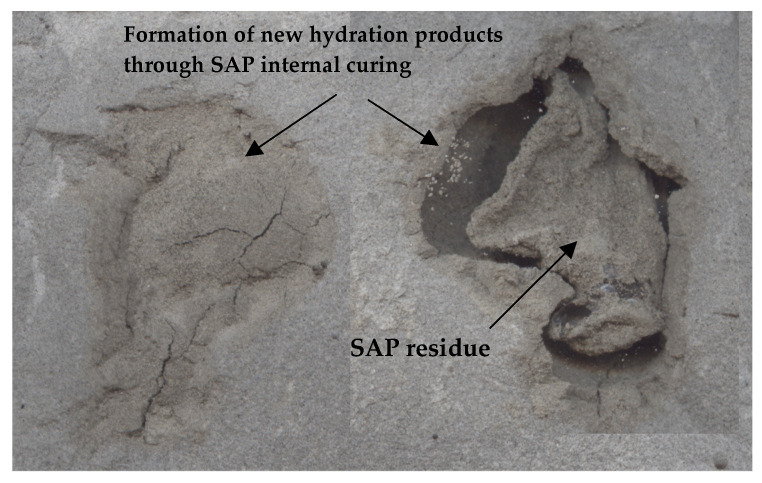
Images of the cement paste with 0.3% wet SAP and a w/c ratio of 0.4 cured for 28 days under Curing 1 conditions (W-26A).

From Figure 13, it can be seen that the addition of 0.3% wet SAP particles caused them to clump together, forming large voids in the cement paste. Under dry curing conditions (Curing 1), internal curing by SAPs can be clearly observed. The internal curing water provided by SAPs assisted in the formation of hydration products, which partially filled the voids created by the SAPs. However, even after 28 days, the water supplied by the SAPs was insufficient to fully fill the voids. Additionally, the SAPs did not fully integrate with the cement paste, leaving behind dried residue. Similarly, in a study conducted by Yu et al. (2019), it was observed that the volume of SAP decreases after water is released, and the water provided by SAP is insufficient to occupy the entire pore space. Thus, the presence of a large number of these pores ultimately led to a reduction in strength [34].

**Figure 14:**
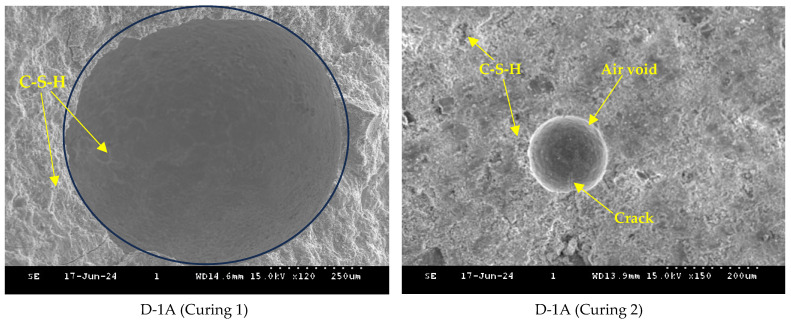

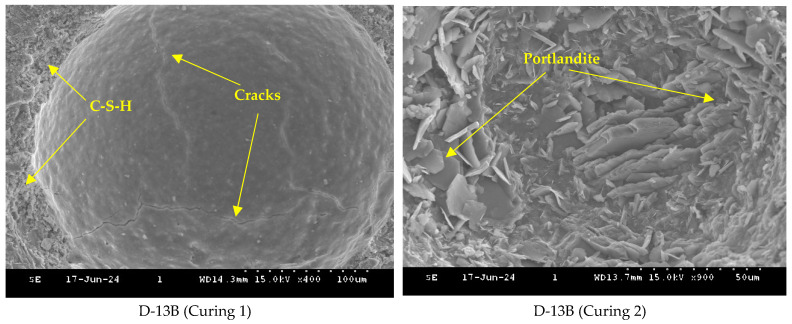
SEM images of cement paste with 0.2% dry SAP and a w/c ratio of 0.4.

**Figure 15:**
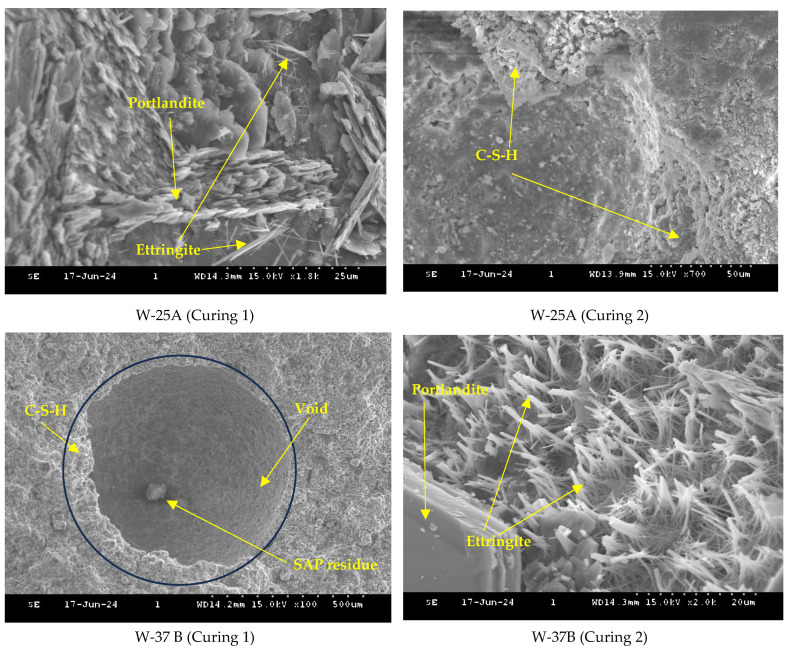
SEM images of cement paste with 0.2% wet SAP and a w/c ratio of 0.4.

Figure 14 and Figure 15 show SEM images of cement pastes with 0.2% dry and wet SAPs and a w/c ratio of 0.4 cured for 28 days under Curing 1 and 2 conditions. The water provided by the SAPs helped in the formation of needle-like ettringite crystals and flat platelet Portlandite crystals (calcium hydroxide), which were predominantly observed in the voids. In addition, fluffy and fibrous C-S-H products were also present in the mixes with SAPs, similar to the findings of Klemm and Sikora (2013) [35]. The voids created by the SAPs were more irregular in shape compared to air voids. Furthermore, the SEM images revealed that the internal curing water provided by the SAP was insufficient to completely fill the voids with hydration products, although it did reduce pore size, which aligns with [36]. Curing 2 conditions (water curing), along with internal curing by SAPs, proved to be more effective in generating hydration products and filling voids compared to the mixes cured under Curing 1 conditions.

## 4. Conclusions

In this study, an experimental investigation was conducted on cement pastes with w/c ratios of 0.4, 0.5, and 0.6 and varying dosages of wet and dry SAP (0.2–0.5% by weight of cement). Two curing conditions were applied: Curing 1, which involved a temperature of 20 °C and 60% RH, and Curing 2, which involved water curing. The objective was to examine the effect of SAP solely on compressive strength and the air void system. The findings of this study are as follows:

1. An increase in the w/c ratio resulted in a decrease in strength for samples cured under Curing 1 conditions. However, samples with a w/c ratio of 0.4 exhibited higher strength when cured under Curing 2 conditions.
2. The strength of cement pastes with dry SAP and varying w/c ratios was affected by Curing 1 conditions. However, cement pastes with a w/c ratio of 0.4 and a dosage of dry SAP B (0.2% and 0.5%) demonstrated improved compressive strength compared to the reference and other samples. In addition, the combination of dry SAP and Curing 1 conditions increased early strength but had a significant impact on later strength, especially after 14 days in most samples. Curing 2 conditions substantially enhanced strength over time in most samples with dry SAP and varying w/c ratios. Cement pastes containing dry SAP A and B with a w/c ratio of 0.4 and 0.5 exhibited higher strength than the reference. The internal water provided by SAP and external water curing helped improve the strength of the cement paste over time.
3. The impact of the curing conditions on strength was similar for both dry and wet SAP additions. When cement paste with wet SAP was cured under Curing 1 conditions, there was a decrease in compressive strength compared to the reference. However, a few samples (W-37B, W-33A, and W-34A) showed better strength than the reference. Under Curing 2 conditions, cement paste with wet SAP resulted in improved strength when the w/c ratio was 0.4 and 0.5. There was a greater reduction in compressive strength when the w/c ratio was 0.6.
4. When comparing dry SAP and wet SAP samples, the dry SAP samples cured under Curing 2 conditions exhibited a higher increase in strength than the wet SAP samples cured under the same conditions. This indicates that dry SAP and Curing 2 conditions improved strength to a greater extent than the reference sample.
5. Samples with dry SAP generally exhibited higher SSAs compared to samples with wet SAP, indicating finer air voids and more surface area within the cement paste structure. Samples with wet SAP tended to have larger air voids, as indicated by their lower SSAs and higher spacing factors compared to samples with dry SAP. Additionally, the samples cured under Curing 1 conditions showed a higher total air content than the samples cured under Curing 2 conditions.
6. Internal water curing provided by SAP helped in the formation of hydration products such as C-S-H, ettringite, and Portlandite crystals. However, the water provision by SAP was inadequate to completely fill the voids with hydration products, although it reduced the pore size.

## Figures and Tables

**Table 1:** Chemical composition of cement.

**Chemical Composition**	CaO	SiO_2_	Al_2_O_3_	Fe_2_O_3_	MgO
**Ratio in weight (%)**	62.00	20.50	5.11	3.30	2.60

**Table 2:** Mix proportions of cement paste with dry SAP with varying SAP dosages.

	D-1A	D-2A	D-3A	D-4A	D-5A	D-6A	D-7A	D-8A	D-9A	D-10A	D-11A	D-12A
**W/C**	0.4	0.4	0.4	0.4	0.5	0.5	0.5	0.5	0.6	0.6	0.6	0.6
**SAP/C (%)**	0.2	0.3	0.4	0.5	0.2	0.3	0.4	0.5	0.2	0.3	0.4	0.5

**Table 3:** Void analysis data for cement paste with a w/c ratio of 0.4 cured under Curing 1 conditions (temperature of 20 °C and 60% RH).

	Total Air (Air Content)		Air ≤ 1 mm		Specific Surface Area (SSA)		Spacing Factor
Sample	%	Stdv.	(%)	Stdv.	(mm^−1^)	Stdv.	(mm)
Ref 0.4	1.88	0.08	1.61	0.07	66.37	4.74	0.14
D-1A (0.2%)	1.81	0.09	1.53	0.09	77.06	2.29	0.12
D-2A (0.3%)	1.97	0.10	1.97	0.10	83.46	1.31	0.11
D-3A (0.4%)	2.41	0.12	2.41	0.12	85.03	9.63	0.10
D-4A (0.5%)	2.38	0.14	2.33	0.14	81.89	2.58	0.10
D-13B (0.2%)	1.90	0.09	1.73	0.09	67.69	15.20	0.14
D-14B (0.3%)	1.95	0.10	1.91	0.10	83.89	3.48	0.11
D-15B (0.4%)	2.09	0.10	2.04	0.10	86.27	7.35	0.10
D-16B (0.5%)	1.70	0.06	1.56	0.06	76.11	17.08	0.13
W-25A (0.2%)	2.36	0.15	1.47	0.06	50.28	4.16	0.17
W-26A (0.3%)	2.20	0.12	1.28	0.07	38.28	5.10	0.22
W-27A (0.4%)	2.31	0.16	0.72	0.03	24.25	1.17	0.35
W-28A (0.5%)	2.39	0.13	1.20	0.05	33.58	6.75	0.25
W-37B (0.2%)	1.95	0.10	1.95	0.11	53.53	1.23	0.17
W-38B (0.3%)	2.32	0.12	2.16	0.13	54.88	5.59	0.15
W-39B (0.4%)	2.54	0.10	2.00	0.10	56.34	9.44	0.14
W-40B (0.5%)	2.65	0.11	2.27	0.10	56.17	2.54	0.14

**Table 4:** Void analysis data for cement paste with a w/c ratio of 0.4 cured under Curing 2 (water curing) conditions.

	Total Air (Air Content)		Air ≤ 1 mm		Specific Surface Area (SSA)		Spacing Factor
Sample	%	Stdv.	%	Stdv.	(mm^−1^)	Stdv.	(mm)
Ref 0.4	1.66	0.06	1.48	0.06	66.99	1.29	0.14
D-1A (0.2%)	0.95	0.04	0.87	0.04	72.36	2.46	0.17
D-2A (0.3%)	1.29	0.08	1.29	0.08	85.22	6.20	0.13
D-3A (0.4%)	1.12	0.06	1.07	0.06	87.25	3.09	0.13
D-4A (0.5%)	1.43	0.08	1.43	0.08	91.09	8.33	0.11
D-13B (0.2%)	1.07	0.04	0.98	0.04	74.68	16.47	0.16
D-14B (0.3%)	0.86	0.04	0.78	0.04	69.24	2.22	0.18
D-15B (0.4%)	1.50	0.09	1.50	0.09	67.65	1.56	0.15
D-16B (0.5%)	1.20	0.07	0.99	0.08	44.46	7.92	0.25
W-25A (0.2%)	1.40	0.06	1.40	0.06	62.79	1.33	0.11
W-26A (0.3%)	1.34	0.06	0.95	0.04	54.53	3.68	0.16
W-27A (0.4%)	1.25	0.05	1.07	0.05	65.30	4.99	0.13
W-28A (0.5%)	1.31	0.06	1.23	0.07	65.84	0.36	0.16
W-37B (0.2%)	1.47	0.06	1.41	0.07	66.13	3.04	0.15
W-38B (0.3%)	1.49	0.08	1.49	0.09	59.35	1.28	0.17
W-39B (0.4%)	1.38	0.06	1.38	0.06	57.21	3.05	0.18
W-40B (0.5%)	1.83	0.09	1.76	0.09	52.97	2.20	0.18

**Table 5:** Void analysis data for cement paste with a w/c ratio of 0.5 cured under Curing 1 (temperature of 20 °C and 60% RH) conditions.

	Total Air (Air Content)		Air ≤ 1 mm		Specific Surface Area (SSA)		Spacing Factor
Sample	%	Stdv.	%	Stdv.	(mm^−1^)	Stdv.	(mm)
Ref 0.5	3.11	0.12	2.92	0.13	56.31	8.05	0.12
D-5A (0.2%)	3.26	0.16	3.26	0.16	76.62	10.33	0.09
D-6A (0.3%)	2.87	0.13	2.77	0.13	62.23	6.07	0.12
D-7A (0.4%)	2.67	0.13	2.62	0.14	67.80	5.16	0.11
D-8A (0.5%)	2.97	0.16	2.91	0.16	79.05	2.51	0.09
D-17B (0.2%)	2.91	0.13	2.85	0.13	64.00	6.49	0.11
D-18B (0.3%)	3.03	0.18	3.03	0.18	68.23	7.36	0.10
D-19B (0.4%)	2.57	0.11	2.51	0.11	67.31	0.26	0.11
D-20B (0.5%)	2.85	0.15	2.71	0.16	74.84	0.62	0.10
W-29A (0.2%)	3.06	0.13	3.00	0.12	47.84	0.47	0.15
W-30A (0.3%)	3.25	0.14	3.25	0.14	53.29	1.57	0.13
W-31A (0.4%)	3.00	0.13	2.94	0.13	52.58	0.40	0.13
W-32A (0.5%)	3.05	0.14	3.00	0.15	53.45	6.61	0.13
W-41B (0.2%)	3.08	0.13	3.08	0.12	56.82	12.38	0.12
W-42B (0.3%)	3.11	0.12	2.91	0.12	52.04	8.35	0.13
W-43B (0.4%)	3.17	0.12	3.05	0.12	54.55	0.28	0.13
W-44B (0.5%)	3.30	0.14	3.11	0.14	61.27	15.34	0.11

**Table 6:** Void analysis data for cement paste with a w/c ratio of 0.5 cured under Curing 2 (water curing) conditions.

	Total Air (Air Content)		Air ≤ 1 mm		Specific Surface Area (SSA)		Spacing Factor
Sample	%	Stdv.	%	Stdv.	(mm^−1^)	Stdv.	(mm)
Ref 0.5	2.22	0.10	2.22	0.10	72.09	1.97	0.11
D-5A (0.2%)	1.79	0.08	1.79	0.08	70.74	6.01	0.13
D-6A (0.3%)	2.14	0.08	1.90	0.09	66.96	3.72	0.12
D-7A (0.4%)	1.75	0.09	1.75	0.09	83.28	3.39	0.11
D-8A (0.5%)	1.83	0.11	1.83	0.11	90.13	0.01	0.10
D-17B (0.2%)	1.59	0.08	1.59	0.08	71.65	4.40	0.13
D-18B (0.3%)	1.71	0.09	1.71	0.09	72.91	0.76	0.12
D-19B (0.4%)	1.62	0.08	1.62	0.08	78.11	13.15	0.12
D-20B (0.5%)	1.90	0.09	1.80	0.09	73.68	6.73	0.12
W-29A (0.2%)	2.15	0.08	2.07	0.07	55.07	3.85	0.15
W-30A (0.3%)	1.79	0.09	1.73	0.10	48.26	4.24	0.18
W-31A (0.4%)	2.07	0.09	1.94	0.09	42.28	1.35	0.20
W-32A (0.5%)	1.92	0.10	1.39	0.07	38.42	2.52	0.22
W-41B (0.2%)	1.97	0.07	1.97	0.06	46.86	0.20	0.18
W-42B (0.3%)	2.40	0.10	2.29	0.09	62.26	6.01	0.13
W-43B (0.4%)	2.27	0.10	2.27	0.10	63.10	2.91	0.13
W-44B (0.5%)	2.36	0.10	2.36	0.10	66.58	3.39	0.12

**Table 7:** Void analysis data for cement paste with a w/c ratio of 0.6 cured under Curing 1 (temperature of 20 °C and 60% RH) conditions.

	Total Air (Air Content)		Air ≤ 1 mm		Specific Surface Area (SSA)		Spacing Factor
Sample	%	Stdv.	%	Stdv.	(mm^−1^)	Stdv.	(mm)
Ref 0.6	3.21	0.18	3.04	0.19	68.09	3.27	0.10
D-9A (0.2%)	3.38	0.23	3.33	0.24	87.67	1.05	0.07
D-10A (0.3%)	3.34	0.16	3.11	0.17	82.21	11.14	0.08
D-11A (0.4%)	3.58	0.21	3.43	0.22	86.00	7.82	0.07
D-12A (0.5%)	3.45	0.19	3.41	0.20	84.30	4.99	0.08
D-21B (0.2%)	3.30	0.20	3.19	0.21	80.11	6.78	0.08
D-22B (0.3%)	3.12	0.17	3.02	0.18	76.76	7.62	0.09
D-23B (0.4%)	3.99	0.28	3.90	0.29	88.57	6.24	0.07
D-24B (0.5%)	3.18	0.18	3.08	0.19	80.04	12.48	0.08
W-33A (0.2%)	3.22	0.15	2.91	0.15	55.91	1.23	0.12
W-34A (0.3%)	3.31	0.15	3.24	0.16	66.44	5.91	0.10
W-35A (0.4%)	3.58	0.17	3.53	0.17	64.51	6.22	0.10
W-36A (0.5%)	3.98	0.21	3.93	0.23	73.14	4.60	0.08
W-45B (0.2%)	3.91	0.20	3.91	0.21	64.49	5.75	0.09
W-46B (0.3%)	3.46	0.15	3.40	0.15	61.94	8.04	0.10
W-47B (0.4%)	3.85	0.19	3.78	0.20	65.34	2.37	0.09
W-48B (0.5%)	4.06	0.23	4.06	0.76	73.52	1.12	0.08

**Table 8:** Void analysis data for cement paste with a w/c ratio of 0.6 cured under Curing 2 (water curing) conditions.

	Total Air (Air Content)		Air ≤ 1 mm		Specific Surface Area (SSA)		Spacing Factor
Sample	%	Stdv.	(%)	Stdv.	(mm^−1^)	Stdv.	(mm)
Ref 0.6	2.37	0.11	2.31	0.11	73.99	16.15	0.10
D-9A (0.2%)	2.31	0.13	2.26	0.13	75.66	11.75	0.10
D-10A (0.3%)	2.58	0.13	2.58	0.13	75.06	12.62	0.10
D-11A (0.4%)	2.19	0.13	2.19	0.13	67.38	6.84	0.12
D-12A (0.5%)	2.22	0.11	2.22	0.11	69.35	12.65	0.11
D-21B (0.2%)	2.49	0.12	2.41	0.12	67.07	5.59	0.11
D-22B (0.3%)	2.62	0.13	2.62	0.13	70.21	11.75	0.10
D-23B (0.4%)	2.95	0.15	2.95	0.15	72.63	3.03	0.09
D-24B (0.5%)	2.20	0.12	2.20	0.12	64.68	0.41	0.12
W-33A (0.2%)	2.80	0.13	2.80	0.13	63.71	6.63	0.11
W-34A (0.3%)	2.89	0.12	2.75	0.12	56.49	0.03	0.12
W-35A (0.4%)	2.72	0.11	2.64	0.11	54.94	3.59	0.13
W-36A (0.5%)	3.19	0.12	2.96	0.12	60.80	6.60	0.11
W-45B (0.2%)	2.93	0.13	2.71	0.13	52.74	0.48	0.13
W-46B (0.3%)	2.85	0.12	2.85	0.11	55.90	0.53	0.12
W-47B (0.4%)	3.17	0.15	3.17	0.15	60.23	0.08	0.11
W-48B (0.5%)	3.10	0.13	3.00	0.13	55.67	1.66	0.12

**Table 9:** ANOVA analysis results for air void analysis of cement pastes with dry and wet SAPs (0.2–0.5%) and a w/c ratio of 0.4, cured under Curing 1 conditions.

SUMMARY						
Groups	Count	Sum	Average	Variance		
Total air (%)	17	36.81	2.17	0.08		
Air ≤ 1 mm (%)	17	30.14	1.77	0.20		
Specific Surface Area (SSA) (mm^−1^)	17	1075.08	63.24	376.70		
Spacing factor (mm)	17	2.64	0.16	0.0042		
ANOVA						
Source of Variation	SS	df	MS	F	*p*-value	F crit
Between Groups	48,852.94	3	16,284.31	172.79	0.00	2.75
Within Groups	6031.718	64	94.25			
Total	54,884.66	67				

SS—Sum of Squares, df—Degree of freedom, MS—Mean square, F—F statistic, F crit—F critical.

**Table 10:** ANOVA analysis on the data of air void analysis of cement pastes with dry and wet SAPs (0.2–0.5%) and a w/c ratio of 0.5 cured under Curing 1 conditions.

SUMMARY						
Groups	Count	Sum	Average	Variance		
Total air (%)	17	51.26	3.02	0.04		
Air ≤ 1 mm (%)	17	49.92	2.94	0.04		
Specific Surface Area (SSA) (mm^−1^)	17	1048.23	61.66	89.04		
Spacing factor (mm)	17	1.98	0.12	0.00026		
ANOVA						
Source of Variation	SS	df	MS	F	*p*-value	F crit
Between Groups	45,440.29	3	15,146.76	679.80	0.00	2.75
Within Groups	1425.993	64	22.28			
Total	46,866.28	67				

**Table 11:** ANOVA analysis on the data of air void analysis of cement pastes with dry and wet SAPs (0.2–0.5%) and a w/c ratio of 0.4 cured under Curing 2 conditions.

SUMMARY						
Groups	Count	Sum	Average	Variance		
Total air (%)	17	22.55	1.33	0.06		
Air ≤ 1 mm (%)	17	21.08	1.24	0.07		
Specific Surface Area (SSA) (mm^−1^)	17	1143.06	67.24	153.25		
Spacing factor (mm)	17	2.66	0.16	0.0011		
ANOVA						
Source of Variation	SS	df	MS	F	*p*-value	F crit
Between Groups	56,112.22	3	18,704.07	487.76	0.00	2.75
Within Groups	2454.18	64	38.35			
Total	58,566.4	67				

**Table 12:** ANOVA analysis on the data of air void analysis of cement pastes with dry and wet SAPs (0.2–0.5%) and a w/c ratio of 0.5 cured under Curing 2 conditions.

SUMMARY						
Groups	Count	Sum	Average	Variance		
Total air (%)	17	33.48	1.97	0.06		
Air ≤ 1 mm (%)	17	32.23	1.90	0.07		
Specific Surface Area (SSA) (mm^−1^)	17	1102.38	64.85	209.41		
Spacing factor (mm)	17	2.37	0.14	0.0012		
ANOVA						
Source of Variation	SS	df	MS	F	*p*-value	F crit
Between Groups	51,465.46	3	17,155.15	327.47	0.00	2.75
Within Groups	3352.777	64	52.39			
Total	54,818.24	67				

## Data Availability

Data are contained within the article.
